# Exploring Thyrotoxic Hypercalcaemia Through Two Cases: A Myriad of Uncertainties

**DOI:** 10.1155/crie/3906797

**Published:** 2026-09-21

**Authors:** Don Asiri Abeysiriwardana, Pahala Gamage Nipuna Jeewantha Dias, Warsha Dilhani De Zoysa, Arosha Sampath Dissanayake

**Affiliations:** ^1^ National Hospital Galle, Ministry of Health, Galle, Sri Lanka, health.gov.lk; ^2^ Department of Medicine, Faculty of Medicine, University of Ruhuna, Galle, Sri Lanka, ruh.ac.lk

**Keywords:** hypercalcaemia, hyperphosphataemia, multinodular goitre, osteoporosis, thyrotoxicosis

## Abstract

Hypercalcaemia is a recognised complication of thyrotoxicosis usually related to increased bone resorption. However, coexistent hyperphosphataemia is uncommon and may create diagnostic uncertainty. It is postulated that serum calcium and phosphate levels rise due to high bone turnover and increased renal reabsorption of phosphate in response to excess thyroxine. Hypercalcaemia may also alter the clinical presentation of thyrotoxicosis, making both diagnosis and management challenging. We report two patients with this unusual biochemical presentation. The first patient was a 69‐year‐old woman who presented with body aches, lethargy, low mood, constipation, polyuria, and proximal muscle weakness for 2 months. The second was a 56‐year‐old woman who presented with palpitations, anxiety, tremulousness, dyspnoea, dysphagia, and headache for 4 months. Both had multinodular goitres (MNGs) without eye disease. Their TSH levels were undetectably low with extremely high free T4 levels. Serum calcium and phosphate levels were high in both patients. ALP levels were mildly elevated, vitamin D levels were deficient or insufficient, and parathyroid hormone (PTH) levels were low‐normal. DXA scans revealed severe osteoporosis. Both patients improved clinically and biochemically with adequate intravenous hydration, frusemide, carbimazole, propranolol, and bisphosphonates. The second patient underwent a successful left thyroid lobectomy, while the first patient remained well on medical treatment. Unexplained hypercalcaemia and hyperphosphataemia should alert the clinicians to the possibility of underlying thyrotoxicosis. Prompt recognition and management is essential to prevent bone loss, correct metabolic abnormalities, and avoid complications. Clinicians should also consider the possibility of hypercalcaemia associated with thyroid dysfunction in patients presenting with nonspecific symptoms such as fatigue, constipation, proximal weakness, polyuria, or mood changes.

## 1. Introduction

Hypercalcaemia is a frequently encountered biochemical abnormality with a wide range of underlying causes. A useful approach to narrowing the differential diagnosis is to consider concurrent phosphate levels. Hypercalcaemia with hypophosphataemia often suggests parathyroid hormone (PTH)–mediated conditions such as primary hyperparathyroidism, familial hypocalciuric hypercalcaemia, or malignancy‐related PTHrP secretion [1, 2]. In contrast, hypercalcaemia with hyperphosphataemia is a less common and often unexpected biochemical pairing, typically associated with vitamin D intoxication, granulomatous disorders such as sarcoidosis and tuberculosis, or osteolytic malignancies like multiple myeloma [1, 3].

Thyrotoxicosis, defined as an excess of circulating thyroid hormones, is a multisystem disorder that can present with a wide spectrum of clinical manifestations, including weight loss, palpitations, tremors, anxiety, and heat intolerance [4, 5]. While mild hypercalcaemia is known to be associated with thyrotoxicosis (often with normal or low phosphate levels), moderate to severe hypercalcaemia coexisting with hyperphosphataemia is particularly unusual with thyroid dysfunction [6–8]. This biochemical profile may therefore mislead clinicians, prompting investigations into alternative diagnoses such as malignancy or granulomatous disease, especially in the absence of overt thyrotoxic symptoms.

The pathogenesis of hypercalcaemia in thyrotoxicosis is multifactorial and is primarily related to increased bone turnover. Excess thyroid hormones enhance osteoclastic activity through elevated interleukin‐6 (IL‐6) levels and heightened bone responsiveness to triiodothyronine (T3), resulting in increased calcium and phosphate release from the bone [9, 10]. Additionally, thyroid hormone excess may alter renal tubular handling of phosphate, leading to reduced phosphate excretion [11]. Suppression of PTH secretion due to hypercalcaemia may further enhance phosphate retention by reducing PTH‐mediated phosphaturia. However, these mechanisms are often underrecognized in clinical practice.

The clinical diagnosis of thyrotoxicosis can be even more challenging when its presentation is masked by symptoms attributable to hypercalcaemia, particularly in older adults who may exhibit features such as lethargy, muscle weakness, constipation, or mood changes. These symptoms can be misattributed to depressive or somatoform disorders if thyroid dysfunction is not considered early [12].

This report describes two patients with thyrotoxicosis manifesting with moderate to severe hypercalcaemia and coexisting hyperphosphataemia, an unusual biochemical combination. Both patients were evaluated and managed at the professorial medical unit of National Hospital of Galle, Sri Lanka. Case 1 was first assessed in December 2024, and Case 2 was first assessed in April 2021. These cases highlight the importance of including thyroid dysfunction in the differential diagnosis of hypercalcaemia, especially when typical aetiologies are not evident.

## 2. Case 1

A 69‐year‐old woman with a history of well‐controlled hypertension and dyslipidaemia presented with generalised body aches, weakness, increased sleepiness, lethargy, abdominal pain, constipation, and polyuria for 2 months. She also had a low mood without any psychotic symptoms, which was being treated as depression with sertraline. She was also on atorvastatin, losartan, and diltiazem. She was not on any other medications, such as vitamins or supplements.

On examination, she appeared ill and dehydrated. She was afebrile and did not have pallor, icterus, or lymphadenopathy. She had a goitre without evidence of thyroid eye disease.

Her pulse was regular, with a rate of 90 beats per minute. Blood pressure was 130/80 mmHg.

There was epigastric tenderness on abdominal examination without organomegaly or free fluid. Digital rectal examination revealed hard stools. Her neurological examination was unremarkable except for grade 3 symmetrical proximal muscle weakness of upper and lower limbs, which impaired her activities of daily living. The rest of the clinical examination was normal.

Complete blood count revealed WBC of 6.3 × 10^9^/L, neutrophils at 60.7%, haemoglobin of 11.1 g/dL, MCV of 86, and platelets of 208 × 10^9^/L. Her ESR was 32 mm/h, with a CRP of 10 mg/L. Thyroid function tests indicated an undetectable TSH level (<0.005 mIU/L) and markedly elevated free T4 (>7.77 ng/dL). Biochemical studies showed high serum corrected calcium levels of 3.2 mmol/L (2.15–2.57), high fasting phosphate levels of 1.9 mmol/L (0.81–1.45), slightly elevated ALP levels of 129 U/L, deficient 25‐hydroxy vitamin D levels of 46 nmol/L, and low‐normal intact PTH levels of 14.5 pg/mL (7.5–53.5).

Her liver profile, renal function tests, blood picture, and serum protein electrophoresis were normal. HbA1c was 5.5%. Thyroid ultrasound revealed a diffusely enlarged gland with multiple nodules, consistent with multinodular goitre (MNG) in the background of thyroiditis. CECT scan of the chest, abdomen, and pelvis did not show evidence of malignancy. No lytic or sclerotic lesions were seen in X‐ray skeletal survey. DXA scan revealed severe osteoporosis.

She was treated with 0.9% NaCl infusion to manage hypercalcaemia. During the hydration process, she developed symptoms of fluid overload, necessitating the use of intravenous frusemide. Propranolol (40 mg twice daily) and carbimazole (20 mg three times daily) were started to treat her thyrotoxicosis. Intravenous zoledronic acid was administered to treat both hypercalcaemia and severe osteoporosis. Serum calcium and phosphate levels normalized within 48 h of the above interventions. Total thyroidectomy was planned for her toxic MNG. All her symptoms had improved by the time of discharge, making her independent in her activities of daily living.

At 1‐year follow‐up, thyroidectomy had not been performed. She remained clinically and biochemically well controlled on antithyroid medication alone, and continued medical management was pursued. At the time of manuscript preparation, repeat DXA assessment had not yet been performed.

## 3. Case 2

A 56‐year‐old woman presented with episodic palpitations, anxiety, chest discomfort, exertional dyspnoea, tremulousness, dysphagia, and worsening headache for 4 months. Her past medical history was significant only for diabetes mellitus and dyslipidaemia. Her only routine medications were metformin and atorvastatin. On general examination, she looked anxious and had a MNG without Graves ophthalmopathy. Her pulse was regular, with a rate of 92 beats per minute. Blood pressure was 120/70 mmHg. There were no other abnormalities on further clinical examination.

Complete blood count had WBC of 7.6 × 10^9^/L, haemoglobin of 11.2 g/dL, and platelets of 208 × 10^9^/L. On initial biochemical assessment, she was found to have hypercalcaemia (serum corrected calcium level—2.82 mmol/L) with hyperphosphataemia (serum fasting phosphate level—1.78 mmol/L). ALP level was 135 U/L, which was slightly high. The TSH level was noted to be undetectably low (<0.005 mIU/L) with a very high free T4 level of >7.77 ng/dL. 25‐Hydroxy vitamin D levels were in the insufficient range (76 nmol/L), and intact PTH levels were low‐normal (10.7 pg/mL).

ESR was 32 mm/h. Her liver enzymes, liver function tests, renal function tests, blood picture, and serum protein electrophoresis were all normal. Fasting blood glucose level was 107 mg/dL. Her cardiac assessment using ECG, troponin I, echocardiography, and 24‐h Holter was unremarkable. Ultrasound scan of the neck revealed MNG with background evidence of thyroiditis. CECT brain and sinuses followed by MRI did not show any abnormality. There was no upper gastrointestinal endoscopic evidence of malignancy even though she complained of dysphagia. X‐ray skeletal survey did not show any lesions. A DXA scan confirmed severe osteoporosis.

Hypercalcaemia was corrected with initial fluid resuscitation, followed by loop diuretics due to circulatory overload. Titrating doses of propranolol and carbimazole were used to control thyrotoxicosis. Osteoporosis was treated with weekly alendronate. After achieving euthyroid status with antithyroid treatment, her corrected serum calcium normalised to 2.53 mmol/L before surgery.

The surgical team elected to perform a left thyroid lobectomy because the dominant nodular burden was located in the left lobe. This limited surgical approach was intended to reduce the risk of permanent postoperative hypoparathyroidism and preserve endogenous thyroid function, thereby potentially avoiding lifelong thyroid hormone replacement. Histology showed colloid goitre with mild lymphocytic thyroiditis, without evidence of malignancy. There was no documented postoperative hypocalcaemia or clinical concern for hungry bone syndrome. At 1‐year follow‐up, she remained clinically well with normal thyroid function, normal phosphate, and normocalcaemia: serum ionised calcium 1.24 mmol/L (reference range 1.15–1.32), fasting phosphate 1.38 mmol/L (reference range 0.75–1.50), TSH 1.73 mIU/L, and free T4 1.41 ng/dL. As of the time of writing, she has not undergone repeat DXA scan for assessment of response to therapy.

## 4. Discussion

In both cases, the key biochemical triad was thyrotoxicosis, hypercalcaemia, and hyperphosphataemia. In the absence of renal impairment, vitamin D excess, granulomatous disease, myeloma, or radiological evidence of malignancy, thyrotoxicosis was considered the most likely unifying explanation for the biochemical abnormalities. However, the absence of urinary calcium and phosphate measurements limits complete exclusion of alternative metabolic contributors. The biochemical improvement after treatment further supported thyrotoxicosis as the major driver of hypercalcaemia.

The coexistence of hyperphosphataemia with thyrotoxic hypercalcaemia is less commonly recognised than hypercalcaemia alone. In thyrotoxicosis, accelerated bone turnover can increase the efflux of both calcium and phosphate from the bone. In addition, hypercalcaemia suppresses PTH secretion, thereby reducing the phosphaturic effect of PTH at the renal tubule. This reduction in PTH‐mediated phosphate excretion may contribute to phosphate retention despite preserved renal function. Thyroid hormone excess may also directly influence renal tubular phosphate handling, although this mechanism is less well established. These combined skeletal and renal mechanisms may explain why phosphate was elevated in both patients despite normal renal function and the absence of vitamin D excess. However, because urinary calcium and phosphate excretion were not measured, the exact contribution of altered renal handling could not be confirmed in our patients.

Free T3 was not available because of local laboratory limitations at the time of assessment. The diagnosis was therefore based on suppressed TSH, markedly elevated free T4 MNG on imaging, and biochemical response to antithyroid treatment. The clinical and biochemical findings, considered together with thyroid ultrasonography in both patients and histopathological findings in Case 2, supported a working diagnosis of toxic MNG with coexisting background thyroiditis. However, because thyroid receptor antibody testing and radionuclide scintigraphy were unavailable, the precise aetiology could not be definitively established.

Both patients had very high free T4 levels in excess of 7.77 ng/dL. Despite markedly elevated thyroid hormone levels, their clinical presentations were not straightforward. The first patient had features of depression and was already on treatment. The second had some typical features of thyrotoxicosis but had prominent atypical features such as headache and dysphagia. We suggest that hypercalcaemia may have played an important role in modifying symptoms, causing a delay in recognition of the thyrotoxic illness. In older adults, nonspecific symptoms and hypercalcaemia may prompt extensive evaluation for malignancy or other systemic disease unless thyroid dysfunction is considered early.

Management should address both acute hypercalcaemia and the underlying thyroid hormone excess. Initial treatment includes intravenous hydration with careful monitoring of volume status. Loop diuretics should not be used routinely for calciuresis but may be required when fluid overload develops, as occurred in these patients. Bisphosphonates may be useful when significant hypercalcaemia coexists with osteoporosis.

Although both patients had severe osteoporosis on baseline DXA assessment and received bisphosphonate therapy, a repeat DXA assessment had not yet been performed at the time of manuscript preparation. Therefore, objective assessment of interval change in bone mineral density was not available.

## 5. Conclusion

Clinicians need to maintain a high index of suspicion for thyrotoxicosis in patients presenting with unexplained hypercalcaemia, particularly when associated with hyperphosphataemia and normal PTH levels. Measuring TSH and serum ionised calcium is essential in patients with nonspecific symptoms such as fatigue, myalgia, constipation, or mood disturbances. Such simple investigations can uncover significant endocrine pathology and prevent misdiagnosing such patients as having primary psychiatric or functional disorders. The combination of thyrotoxicosis with hypercalcaemia presents a myriad of uncertainties to the clinician.

## Funding

This work did not receive any specific grant from any funding agency in the public, commercial, or not‐for‐profit organisations.

## Disclosure

All authors have read and approved the final version for submission for publication. Don Asiri Abeysiriwardana had full access to all of the data in this study and takes complete responsibility for the integrity of the data and the accuracy of the data analysis. The authors critically reviewed and edited all AI‐assisted output and take full responsibility for the accuracy, integrity, and final content of the manuscript.

## Ethics Statement

This report was prepared using deidentified clinical information. Formal ethics approval was not required according to local institutional requirements.

## Consent

Informed written consent has been obtained for the publication.

## Conflicts of Interest

The authors declare no conflicts of interest.

## Data Availability

The data supporting the findings of this report are available from the corresponding author upon reasonable request, subject to institutional and patient confidentiality requirements.

## References

[1] Tebben P. J. , Singh R. J. , and Kumar R. , Vitamin D-Mediated Hypercalcemia: Mechanisms, Diagnosis, and Treatment, Endocrine Reviews. (2016) 37, no. 5, 521–547, 10.1210/er.2016-1070. PMC5045493 27588937

[2] Sadiq N. M. , Anastasopoulou C. , Patel G. , and Badireddy M. , Hypercalcemia, StatPearls [Internet], 2026, StatPearls Publishing, Treasure Island (FL). 28613465

[3] Jacobs T. P. and Bilezikian J. P. , Rare Causes of Hypercalcemia, The Journal of Clinical Endocrinology & Metabolism. (2005) 90, no. 11, 6316–6322, 10.1210/jc.2005-0675. 16131579

[4] Unachukwu C. N. , Chinenye S. , and Uchenna D. I. , Thyrotoxicosis—A Review, Port Harcourt Medical Journal. (2008) 2, no. 3, 10.4314/phmedj.v2i3.38920.

[5] Franklyn J. A. and Boelaert K. , Thyrotoxicosis, The Lancet. (2012) 379, no. 9821, 1155–1166, 10.1016/S0140-6736(11)60782-4. 22394559

[6] Chen K. , Xie Y. , Zhao L. , and Mo Z. , Hyperthyroidism-Associated Hypercalcemic Crisis: A Case Report and Review of the Literature, Medicine. (2017) 96, no. 4, 10.1097/MD.0000000000006017. PMC5287984 28121960

[7] Mosekilde L. , Eriksen E. F. , and Charles P. , Effects of Thyroid Hormones on Bone and Mineral Metabolism, Endocrinology and Metabolism Clinics of North America. (1990) 19, no. 1, 35–63, 10.1016/S0889-8529(18)30338-4. 2192868

[8] Greenspan S. L. and Greenspan F. S. , The Effect of Thyroid Hormone on Skeletal Integrity, Annals of Internal Medicine. (1999) 130, no. 9, 750–758, 10.7326/0003-4819-130-9-199905040-00016. 10357695

[9] Khan H. , Nawaz M. , Schofield J. , and Soran H. , Severe Hypercalcaemia Secondary to Relapsed Graves’ Disease, British Medical Journal Case Reports. (2021) 14, no. 1, 10.1136/bcr-2020-238898. PMC7816925 33462034

[10] Iqbal A. A. , Burgess E. H. , Gallina D. L. , Nanes M. S. , and Cook C. B. , Hypercalcemia in Hyperthyroidism: Patterns of Serum Calcium, Parathyroid Hormone, and 1,25-Dihydroxyvitamin D3 Levels During Management of Thyrotoxicosis, Endocrine Practice. (2003) 9, no. 6, 517–521, 10.4158/EP.9.6.517. 14715479

[11] Yamashita H. , Yamazaki Y. , and Hasegawa H. , et al.Fibroblast Growth Factor-23 in Patients With Graves’ Disease Before and After Antithyroid Therapy: Its Important Role in Serum Phosphate Regulation, The Journal of Clinical Endocrinology & Metabolism. (2005) 90, no. 7, 4211–4215, 10.1210/jc.2004-2498. 15827108

[12] Parker K. I. , Loftley A. , Charles C. , and Hermayer K. L. , A Case of Apathetic Thyroid Storm With Resultant Hyperthyroidism-Induced Hypercalcemia, The American Journal of the Medical Sciences. (2013) 346, no. 4, 338–340, 10.1097/MAJ.0b013e31828ffcbc. 23608928

